# Comparison of Postoperative Port-Site Pain After Gallbladder Retrieval From Epigastric Versus Umbilical Port in Patients of Laparoscopic Cholecystectomy for Symptomatic Cholelithiasis: A Randomized Controlled Trial

**DOI:** 10.7759/cureus.18087

**Published:** 2021-09-18

**Authors:** Arihant Jain, Muhamed Tajudeen, Amith Sreekanth, Nagarajan Raj Kumar

**Affiliations:** 1 Surgery, Jawaharlal Institute of Postgraduate Medical Education & Research, Puducherry, IND

**Keywords:** port-site pain, epigastric port, umbilical port, postoperative pain, laparoscopic cholecystectomy

## Abstract

Introduction

Gallbladder (GB) retrieval is an important cause of postoperative pain (POP) after laparoscopic cholecystectomy (LC). Retrieval is through the epigastric or umbilical port based on the surgeon’s preference. There is limited evidence to support the superiority of one port over the other in terms of POP. This study was done to compare POP between epigastric and umbilical ports after GB retrieval in LC for symptomatic cholelithiasis.

Material and methods

All patients who underwent elective LC for symptomatic cholelithiasis were randomized for GB retrieval either through the umbilical (n = 15) or epigastric (n = 15) port. Postoperatively, the retrieval difficulty score by the operating surgeon, visual analog scale (VAS) scores for pain, and surgical site infection (SSI) by postoperative day (POD) 10 and 30 were assessed.

Results

The mean visual analog scores at the umbilical port at 1, 6, 12, 24, and 36 hours postoperatively were 5.20 ± 0.86, 4.60 ± 0.74, 4.00 ± 0.53, 3.40 ± 0.08, and 2.73 ± 0.82, which were significantly less than the visual analog scores at the epigastric port at the same time intervals, measuring 6.06 ± 1.34, 5.87 ± 1.30, 5.27 ± 1.16, 4.73 ± 1.10, and 3.93 ± 1.03, respectively. The difference was statistically significant between the two arms (p-value < 0.05). The mean retrieval difficulty score was significantly less for the umbilical port (4.40 ± 0.74) when compared with the epigastric port (5.13 ± 0.55). The overall SSI rate in the present study was 10%, and three (20%) patients in the epigastric port group developed SSI by POD 10, while none in the umbilical port group developed SSI.

Conclusion

GB retrieval from the umbilical port is associated with less POP, SSI, and retrieval difficulty when compared with GB retrieval from the epigastric port after elective LC for symptomatic cholelithiasis. Titration of analgesic use can also be done appropriately, reducing the dose of analgesics after 12-24 hours.

## Introduction

Laparoscopic cholecystectomy (LC) is the gold standard for the treatment of benign gallbladder (GB) disease such as symptomatic cholelithiasis. In LC, the retrieval of the GB is either via the umbilical or epigastric ports as per the surgeon's preference [1-3]. LC has completely transformed GB surgery as it is associated with less postoperative pain (POP) and decreased risk of surgical site infection (SSI) compared to open cholecystectomy [1,4,5]. LC has advantages of shorter hospital stay and early recovery and is cost-effective [6]. POP is an important morbidity after LC [7]. GB retrieval is one of the factors described for POP [8]. There is a paucity of data to establish the superiority of one port over the other for GB retrieval with regard to POP [1]. Hence, this study was done to compare POP between epigastric and umbilical port after GB retrieval after LC for benign GB disease.

## Materials and methods

Trial design

This study is a single-center, prospective, parallel-arm, single-blinded, randomized control trial (RCT) comparing port-site pain after LC. The study was conducted in the Department of Surgery, Jawaharlal Institute of Postgraduate Medical Education & Research (JIPMER), for a period of three months between August 2019 and October 2019 after approval by the Institute Ethics Committee (IEC). The study was registered at the Clinical Trials Registry - India (Reg. No.: CTRI/2019/08/020795).

Participants

All consecutive patients, aged 18 years and above, who were in the outpatient follow-up for symptomatic cholelithiasis and admitted for elective LC in the Department of Surgery were included in the study.

The following patients were excluded from the study: patients less than 18 years; adults with acute cholecystitis, empyema of the GB, mucocele of the GB, or suspected/proven malignancy of the GB; patients in whom laparoscopy was converted to open cholecystectomy; and patients who were chronic users of analgesic medicines and steroids.

Interventions

A total of 30 patients were randomized into two groups: (1) the umbilical port group (GB retrieval through the umbilical port) and (2) the epigastric port group (GB retrieval through the epigastric port). Covidien VersaOne Bladeless Trocar with Fixation Cannula (Covidien, Mansfield, USA) was used for all patients.

Preoperative Care

Preoperative preparation was identical in both groups. Patients were admitted the day before surgery. They received similar preanesthetic medication.

Intraoperative Care

LC was done by experienced surgeons who had performed more than 30 LC. All patients underwent LC under general anesthesia. The bladder was catheterized, and a nasogastric tube was inserted in all patients before the procedure. The GB parts were painted with Betadine and surgical spirit and were draped. A prophylactic dose of 1 g of ceftriaxone was given intravenously before skin incision. An open technique with direct visualization (Hasson’s technique) was used for the initial trocar insertion in the supra-umbilical region; pneumoperitoneum was created and maintained at 12 mmHg. LC was done by a standard four-port technique: two 10-mm ports were inserted in the supra-umbilical and epigastric sites; one 5-mm port was inserted in the right hypochondriac region, 2-3 cm below the costal margin in the midclavicular line; and another 5-mm port was inserted in the right flank for the retraction of the GB fundus. The retrograde technique of GB dissection was followed in all patients. The GB specimen was removed via umbilical or epigastric port depending upon the allocation of groups using a retrieval bag. Hemostasis was confirmed. The fascias in the epigastric and umbilical ports were closed with interrupted Prolene 1-0 sutures. The skin was closed with interrupted Prolene sutures/skin staples, and dressing was done.

Postoperative Care

The patients were started on orals six hours after the surgery and escalated to a normal diet. Postoperatively, a nonsteroidal anti-inflammatory drug (NSAID), such as diclofenac 50 mg, was given twice a day till discharge. The wound was inspected for SSI, and the patients were discharged on postoperative day (POD) 2 if there were no complications.

Outcome measures

The primary endpoint was POP at the site of the ports between the two groups. Data of pain scores were collected postoperatively at 1, 6, 12, 24, and 36 hours using the visual analog scale (VAS) in both groups. The secondary endpoints were the difficulty of GB retrieval from the ports and SSI among both groups. Retrieval difficulty (1 being the easiest and 10 being the hardest) was marked by the operating surgeon immediately after the surgery, while the assessment for SSI was done on POD 10 and 30 during the follow-up visits of patients to the hospital according to the CDC guidelines.

Sample size calculation 

Among the patients with symptomatic cholelithiasis, assuming a two-sided 5% significance level, with a power of 80%, the VAS score for pain in the epigastric port retrieval of 3.05 and that in the umbilical port retrieval of 2.15 at 24 hours; the sample size was 15 participants in each group [1]. Then, considering the attrition rate of 20%, the corrected sample size was 18 participants in each group. The sample size was calculated using the Open Epi software version 3.01.

Randomization

The patients were randomly assigned in a 1:1 ratio for retrieving GB via the epigastric port or umbilical port after LC. Block randomization was done using a computer-generated program with randomly selected block sizes of two, four, and six. Allocation concealment was performed using the serially numbered opaque sealed envelope (SNOSE) technique. 

Blinding

This was a single-blinded study. The sequence was generated by a person in the Department of Preventive and Social Medicine, who was not part of the study. Group allocation was done in the operating room during GB retrieval. The principal investigator (PI) was blinded, and data were collected from patients in the postoperative ward and on follow-up visits.

Statistical methods

**Figure 1:**
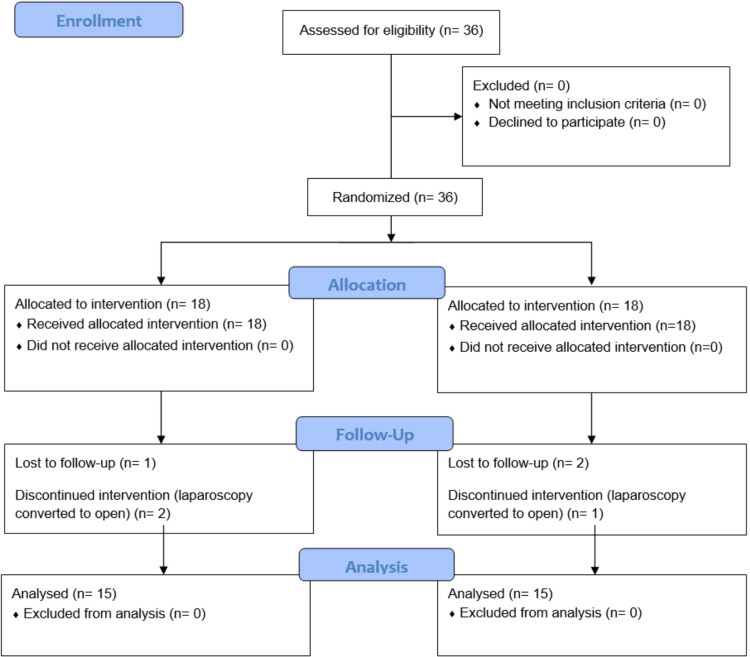
CONSORT diagram of the trial. CONSORT: Consolidated Standards of Reporting Trials

The types of variables used for the analysis include independent variables (age, gender), outcome variables (pain scores, retrieval difficulty scores), confounding variables, and interacting variables (SSI). Data were analyzed using the SPSS software version 20.0 (IBM SPSS Statistics, Armonk, NY). Continuous variables, such as age and duration of surgery, and discrete variables, such as pain scores at 1, 6, 12, 24, and 36 hours, were expressed as mean ± SD. Categorical variables, such as gender and site of GB retrieval, were expressed as percentages or proportions. Mann-Whitney test was used to compare pain scores between the two groups. P-value < 0.05 was considered statistically significant. The study is depicted in line with the Consolidated Standards of Reporting Trials (CONSORT) guidelines, as shown in Figure 1.

## Results

Baseline data 

The present study was conducted from August 2019 to October 2019; 30 patients were randomized into two groups (15 each in the epigastric and umbilical port groups). The mean age was 48.87 ± 9.67 years in the umbilical group and 45.87 ± 12.70 years in the epigastric group. A large proportion of the patients in the study group were females (83.33%). With regard to gender distribution, 12 (80%) out of the 15 subjects in the umbilical port group were females, and 13 (86.66%) out of the 15 subjects in the epigastric port group were females. Gender distribution was comparable in both groups (Table 1). The mean body mass index (BMI) was 23.23 ± 1.01 in the umbilical port group and 22.79 ± 0.92 in the epigastric port group. The mean length of hospital stay was 47 ± 3 hours in the umbilical port group and 46 ± 5 hours in the epigastric port group. The two groups were comparable in terms of all demographic and baseline clinicopathologic characteristics (Table 1).

**Table 1:** Comparison of the baseline variables between the umbilical port group and the epigastric port group. BMI - body mass index

Variables	Umbilical port (n = 15)	Epigastric port (n = 15)
Age (years)	48.87 ± 9.67	45.87 ± 12.70
Sex (male:female)	3:12	2:13
Mean BMI (kg/m^2^)	23.23 ± 1.01	22.79 ± 0.92
Mean length of hospital stays (hours)	47 ± 3	46 ± 5

Outcomes

Comparison of Pain Scores Between the Exit and Non-exit Port

**Figure 2:**
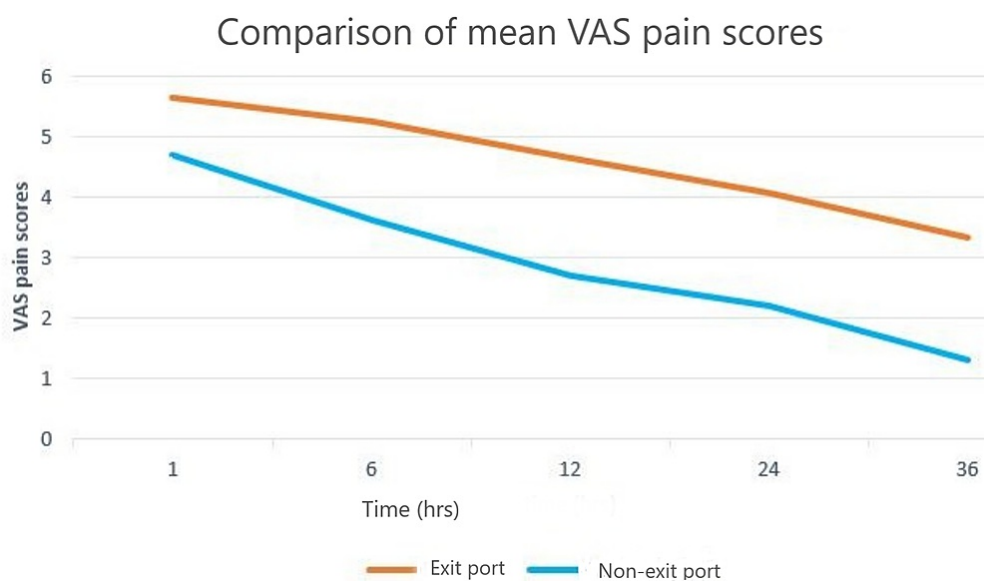
Graphical representation of the mean VAS pain scores of the exit (umbilical/epigastric) port versus non-exit (epigastric/umbilical) port. VAS - visual analog scale

Table 2 and Figure 2 compare the mean VAS pain score at 1, 6, 12, 24, and 36 hours between the exit port through which the GB was removed (either umbilical or epigastric) and the non-exit port. It shows that pain scores were higher for exit port at all time intervals, and the difference was statistically significant (P < 0.01).

**Table 2:** Comparison of mean VAS pain scores for exit (umbilical or epigastric) or non-exit (epigastric or umbilical) port. VAS - visual analog scale SD - standard deviation *Statistically significant at p-value < 0.05

Time interval (hours)	Exit port VAS score (mean ± SD)	Non-exit port VAS score (mean ± SD)	Difference	P-values
1	5.63 ± 1.19	4.70 ± 0.84	0.93	<0.01*
6	5.23 ± 1.22	3.60 ± 0.93	1.63	<0.01*
12	4.63 ± 1.09	2.70 ± 0.83	1.93	<0.01*
24	4.07 ± 1.14	2.20 ± 1.00	1.87	<0.01*
36	3.33 ± 1.06	1.30 ± 0.75	2.03	<0.01*

Comparison of Pain With Relation to Exit Ports

**Figure 3:**
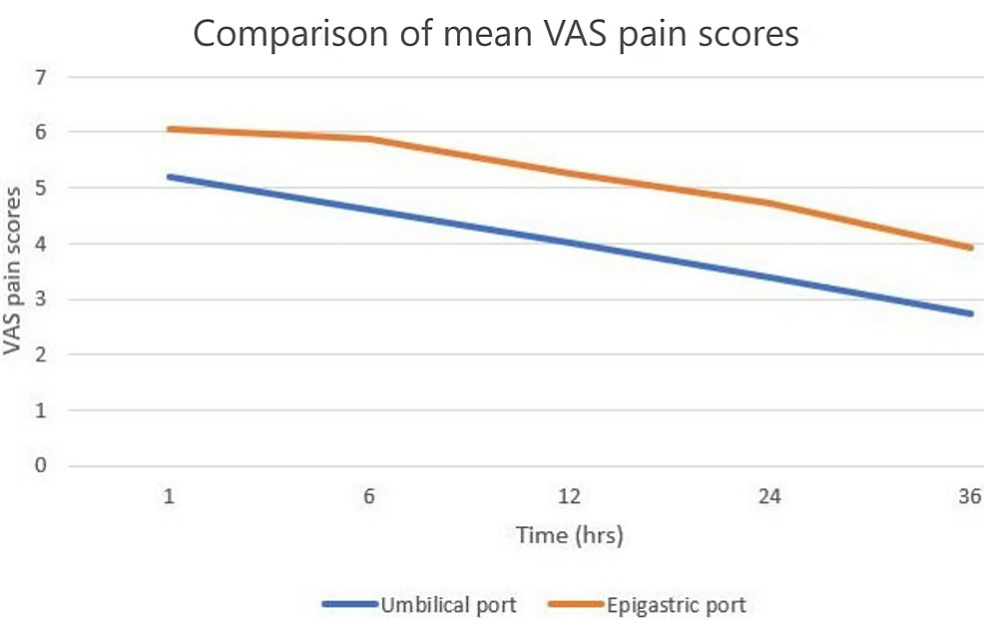
Graphical representation of mean VAS pain scores between the umbilical and epigastric exit ports. VAS - visual analog scale

Table 3 and Figure 3 compare the mean VAS pain scores at 1, 6, 12, 24, and 36 hours between the epigastric port and the umbilical port. Pain scores were less for the umbilical port at all measured periods, which was statistically (P < 0.05) and clinically (difference of equal to or more than 0.8 VAS pain score) significant. The maximum difference in pain score was 1.33 at 24 hours. 

**Table 3:** Comparison of pain scores, retrieval difficulty, and surgical site infection between the umbilical and the epigastric port. SD - standard deviation *Statistically significant at p-value < 0.05

Serial number	Variable		Umbilical port (n = 15) (mean ± SD)	Epigastric port (n = 15) (mean ± SD)	P-value
1	Mean VAS pain scores at time intervals (mean ± SD)	1 hour	5.20 ± 0.86	6.06 ± 1.34	<0.05*
		6 hours	4.60 ± 0.74	5.87 ± 1.30	<0.05*
		12 hours	4.00 ± 0.53	5.27 ± 1.16	<0.05*
		24 hours	3.40 ± 0.08	4.73 ± 1.10	<0.05*
		36 hours	2.73 ± 0.82	3.93 ± 1.03	<0.05*
2	Retrieval difficulty score (mean ± SD)		4.40 ± 0.74	5.13 ± 0.55	<0.05*
3	Surgical site infection on postoperative day 10 (n)		0	3	-

Comparison of Retrieval Difficulty With Relation to the Exit Ports

Table 3 compares the mean retrieval difficulty of the GB for the surgeon between the umbilical and the epigastric port groups. The retrieval difficulty score for the umbilical port was less than that for the epigastric port by 0.73, which was statistically significant (P < 0.05) (4.40 ± 0.74 vs. 5.13 ± 0.55).

Comparison of Infection With Relation to the Exit Ports

Table 3 also compares the incidence of SSI between the umbilical and the epigastric port groups. Three patients (20%) in the epigastric port group had SSI on POD 10, but none of the patients in the umbilical port group had SSI. No patient had SSI on POD 30 in both groups. The overall SSI rate was 10%. 

## Discussion

LC is the treatment method of choice for symptomatic cholelithiasis. LC is associated with lesser POP and lesser incidence of SSI as compared to open cholecystectomy [1,4,5]. Port-site pain is a common morbidity after LC and is the main reason for prolonged in-hospital stay [7]. The extraction of the GB is one of the important factors affecting POP. Currently, the GB is retrieved either from the epigastric or umbilical port based on the surgeon’s preference. 

The present study was conducted among adults with symptomatic cholelithiasis undergoing LC to assess POP (VAS), mean retrieval difficulty, and SSI in GB retrieval from umbilical port compared with epigastric port. Many trials have studied the use of NSAIDs, preemptive analgesia (incisional or intraperitoneal infiltration of local anesthetic agent), intraperitoneal saline, a gas drain, heated gas, low-pressure gas, and nitrous oxide pneumoperitoneum in pain relief after LC, but none has been recommended as the standard of care [1,9,10]. To date, there is no reliable evidence to support any one port being superior to the other for GB extraction while considering the POP and SSI.

The present study showed a female preponderance (83.33%) of GB diseases, similar to previous studies like Shakya et al. (75% females) and Siddiqui et al. (76% females) [1,10]. The maximum number of patients belonged to the age group of 40-49 years. The mean age of patients was 48.87 ± 9.67 years for the umbilical port group and 45.87 ± 12.70 years for the epigastric port group. The mean age of the patients in the study conducted by Siddiqui et al. was 42.5 ± 10.7 years in the umbilical port group and 40.6 ± 12.6 years in the epigastric port group [1]. The mean age of the patients in the present study was similar to the mean age of the study done by Siddiqui et al., and it shows an increased incidence of symptomatic cholelithiasis in the age group of 40-50 years.

The mean BMI of the patients in the umbilical port group was 23.23 ± 1.01 and in the epigastric port group was 22.79 ± 0.92. Visceral and parietal pain are the most important determinants of pain in the first 24-36 hours post-surgery. The pain after LC depends upon multiple factors, including blood vessel injury on incision of the rectus sheath, trauma to the nerve fibers, extraction of the GB, and the pneumoperitoneum created [8].

The present study showed that the pain scores at the exit port from which the GB was removed (either umbilical or epigastric) were higher as compared with the non-exit port, depending upon the patient’s randomization, similar to the previous study conducted by Hajong et al. [11]. This proved the fact that GB retrieval is one of the important factors affecting POP after LC. The higher pain scores may be attributed to the forced stretching of the sheath and muscle, use of dilators, and sometimes skin tears at the time of the retrieval of the GB.

Higher pain scores were observed at all measured time intervals for the epigastric port group as compared with the umbilical port group. The maximum difference in pain score was 1.33 at 24 hours, which was statistically significant (P < 0.05). Similar results were noted by Siddiqui et al., who claimed a difference of 1.5 at 12 hours, and Hajong et al., who claimed a significant difference of 1.5 at 12 hours [1,11]. Since the maximum pain score difference was seen between 12 and 24 hours, analgesics can be reduced in dose or frequency after 12-24 hours postoperatively. The difference in the pain scores between the umbilical port and the epigastric port group was more than or equal to 0.8 on VAS at all measured time intervals, similar to the study conducted by Siddiqui et al., where port-site pain scores were equal to or more than 0.9 (P < 0.01) higher for the epigastric port group than the umbilical port group [1]. Hajong et al. found that port-site pain scores were equal to or more than 1.1 (P < 0.001) higher for the epigastric port group [11]. This could be explained by the fact that the umbilical port is inserted by an open technique, making a 5-mm stab incision on the sheath, which gives wider space for the retrieval of the GB, with less traction of the sheath and parietal peritoneum and therefore less pain, whereas epigastric port is inserted by blunt force, injuring the rectus sheath, leading to more chances of hematoma formation and therefore increased pain. Moreover, the retrieval of the GB through the epigastric port will cause excessive traction on the parietal peritoneum and rectus muscle, inciting the nerve fibers, leading to increased pain perception.

The mean retrieval difficulty was also less for the umbilical port group (4.40 ± 0.74) when compared with the epigastric port group (5.13 ± 0.55), and the difference was statistically significant (P < 0.005). This was because the epigastric port group was inserted at an angle to the abdominal wall compared to vertical insertion, lax sheath, and open technique port insertion at the umbilical region, thereby giving wider space for GB retrieval through the umbilical port. It also depends on the technique of the retrieval and the difference in the surgeon’s perception of rating difficulty. This was contrary to the studies by Siddiqui et al., where the mean retrieval difficulty score was significantly higher for the umbilical port group (4.4 ± 1.2) than that for the epigastric port group (4.2 ± 1.1), and Hajong et al. claimed a significantly prolonged time for GB removal from the umbilical port (4.94 ± 1.56 minutes) when compared with the epigastric port group (3.24 ± 1.29 minutes) [1,11].

The overall rate of SSI in the present study was 10%. Three (20%) of the patients in the epigastric port group developed SSI during follow-up on POD 10, while none of the patients in the umbilical port group developed SSI. A similar result was seen in the study conducted by Shakya et al., where the SSI rate was less for the umbilical port group (3%) in comparison with the epigastric port group (5%) [10]. There are high chances of hematoma formation at the epigastric port site due to the direct incision of the sheath and bile contamination of the port site due to retrieval difficulty, thereby increasing the risk for SSI.

The present study is not without its own limitations. The association of pain with comorbidities was not assessed, and fewer outcome variables were used for the analysis. The sample size of the study is considerably small.

The results of the present study can be applied in the titration of analgesic use. The pain scores were higher for the epigastric group by equal to or more than 0.8 at measured time intervals, which is statistically significant. The maximum pain score difference was 1.33 at 24 hours. Therefore, analgesics can be reduced in dose or frequency after 12-24 hours postoperatively.

## Conclusions

The retrieval of the GB from the umbilical port is superior to that from the epigastric port in terms of decreased POP, less retrieval difficulty, and decreased SSI in patients undergoing LC for benign GB disease. Thus, the authors recommend the routine use of the umbilical port for GB retrieval with appropriate wound protective measures. Titration of analgesic use can also be done appropriately, reducing the dose of analgesics after 12-24 hours.
